# Spark Plasma Sintering of a Gas Atomized Al7075 Alloy: Microstructure and Properties

**DOI:** 10.3390/ma9121004

**Published:** 2016-12-12

**Authors:** Orsolya Molnárová, Přemysl Málek, František Lukáč, Tomáš Chráska

**Affiliations:** 1 Department of Physics of Materials, Faculty of Mathematics and Physics, Charles University, Ke Karlovu 5, Prague 12116, Czech Republic; malek@met.mff.cuni.cz; 2 Institute of Plasma Physics of the CAS, Za Slovankou 1782/3, Prague 18200, Czech Republic; lukac@ipp.cas.cz (F.L.); chraskat@ipp.cas.cz (T.C.)

**Keywords:** gas atomized Al7075 alloy, spark plasma sintering, microstructure, microhardness, high temperature stability

## Abstract

The powder of an Al7075 alloy was prepared by gas atomization. A combination of cellular, columnar, and equiaxed dendritic-like morphology was observed in individual powder particles with continuous layers of intermetallic phases along boundaries. The cells are separated predominantly by high-angle boundaries, the areas with dendritic-like morphology usually have a similar crystallographic orientation. Spark plasma sintering resulted in a fully dense material with a microstructure similar to that of the powder material. The continuous layers of intermetallic phases are replaced by individual particles located along internal boundaries, coarse particles are formed at the surface of original powder particles. Microhardness measurements revealed both artificial and natural ageing behavior similar to that observed in ingot metallurgy material. The minimum microhardness of 81 HV, observed in the sample annealed at 300 °C, reflects the presence of coarse particles. The peak microhardness of 160 HV was observed in the sample annealed at 500 °C and then aged at room temperature. Compression tests confirmed high strength combined with sufficient plasticity. Annealing even at 500 °C does not significantly influence the distribution of grain sizes—about 45% of the area is occupied by grains with the size below 10 µm.

## 1. Introduction

Powder metallurgy represents a very effective tool for processing metallic materials that can achieve properties superior to their ingot metallurgical counterparts. Powder particles can be prepared by various methods. For several decades, atomization techniques have been considered the most important ones. Due to very high cooling rates (up to 10^5^ K·s^−1^) the atomized materials are far from thermodynamic equilibrium—the content of dissolved alloying elements can be enhanced significantly above the equilibrium solid solubility limit and some metastable phases and a fine-grained microstructure can be formed [1,2]. All the above mentioned aspects can contribute to an improvement of mechanical properties.

Powder consolidation is probably the most serious problem of powder metallurgy. The current consolidation methods (e.g., high isostatic pressing) generally require a high temperature and a long sintering time. During this period, the material tends to reach its thermodynamic equilibrium and many gains from atomization can be lost. Much attention has been recently paid to spark plasma sintering/field assisted sintering technology (SPS/FAST) (e.g., [3]). This technique combines applied uniaxial pressure with heating by low voltage pulsed DC current flowing through the sample. High current density and large Joule heat can be evolved at contact points of powder particles where the temperature can highly exceed the set one. Sintering occurs at these points and the interior of the powder particles is nearly unaffected and retains the microstructure formed during atomization. Spark discharge is limited to contact points where it can break up the oxide layers present at the powder particle surface and contributes thus to a very low porosity of the sintered material [4]. A further advantage of SPS is a short time of exposition to elevated temperatures which helps to limit undesirable processes like grain growth.

Gas atomized powders were used for processing of various Al-based alloys. High temperature Al alloys (i.e., alloys preserving their high strength at temperatures up to 400 °C) can be prepared (e.g., [5,6]). The Al7075 alloy processed by extrusion of the atomized powder was successfully tested for superplastic behavior [7,8]. The combination of gas atomization and high pressure cold deposition was used for the preparation of a compact Al7075 alloy [9]. The main problem of these compacts was an inhomogeneity along the direction of deposition. The Al7075 prepared using spray-forming followed by extrusion exhibited extremely high porosity (up to 20 vol %) [10]. A combination of gas atomization and semi-solid rolling was tested at the Al–Zn–Mg–Cu–Zr alloy [11]. Numerous very large pores were present in the rolled material.

Atomized powders can be used as a starting material for further processing, as mechanical milling or mechanical alloying. This processing introduces a large deformation energy into the powder material. Milled powder particles contain a large density of dislocations, intermetallic phases can be dissolved and frequently a nanocrystalline structure can be formed. Asgharzadeh et al. [12] reported a grain size of 100 nm in the milled powder of the AA6063 alloy and a broad distribution of grain sizes between 50 nm and several μm in the hot extruded compact. Hot extrusion resulted in a grain elongation along the extrusion direction and a presence of numerous low-angle boundaries. Mechanical alloying (milling of elemental powders) combined with hot pressing was used for the processing of an Al–Zn–Mg–Cu–Zr alloy [13]. The nanocrystalline microstructure contributed to a very high microhardness of 234 HV despite the presence of 3 to 7 vol % of pores in the hot pressed compact. Similarly, high microhardness values were found also in the mechanically alloyed and hot pressed Al7075 alloy [14]. The lowest porosity (below 1 vol %) was reported by Saheb et al. in mechanically milled and spark plasma sintered Al–SiC nanocomposites [15].

The commercial Al7075 alloy was chosen for our experiments. This alloy is a typical precipitation strengthened material which derives its high strength from the precipitation sequence occurring either at room temperature (natural ageing) or at elevated temperatures (artificial ageing) after solution treatment. The well-known sequence in this type of alloys—supersaturated solid solution—Guinier-Preston zones—metastable η′ phase—stable η-MgZn_2_ phase [16,17] can be modified by chemical composition; presence of microstructure defects, further sites for heterogeneous nucleation (e.g., primary particles rich in Fe or Si) [18], and other complex phases can be formed. It is believed that powder metallurgical processing can improve the mechanical properties of this alloy. The combination of gas atomization and spark plasma sintering represents a first step in our research.

Our work has focused on the following topics:

- To investigate the microstructure of atomized powder particles, especially the distribution of intermetallic phases, and the character of interfaces present in the powder particles’ interior;
- to investigate the influence of SPS on microstructure and phase composition;
- to investigate the microstructural stability of the compact material at elevated temperature;
- to investigate the microhardness of both powder and SPS materials.

This investigation is a basis for further research on mechanically milled material.

## 2. Materials and Methods

**Figure 1:**
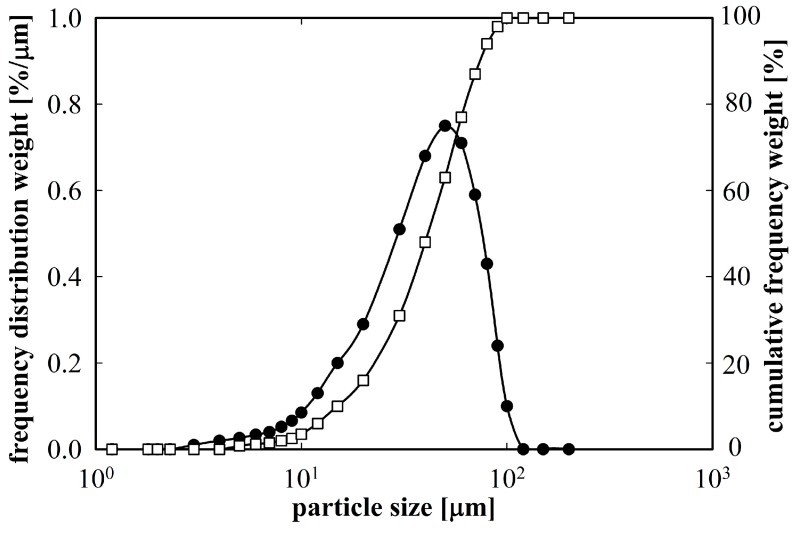
Particle size distribution of the atomized powder material.

The Al7075 alloy was re-melted in silica crucibles of the high energy gas atomizer PSI HERMICA 10/21 VI at 900 °C. The melt was atomized with nitrogen (gas pressure from 3 to 5 MPa, calculated gas velocity 480 m/s). The resulting powder was sieved in order to remove powder particles exceeding 100 µm in size. Figure 1 shows a relatively broad distribution of particle sizes, however, about 90% of the powder material was below 70 µm. 

**Figure 2:**
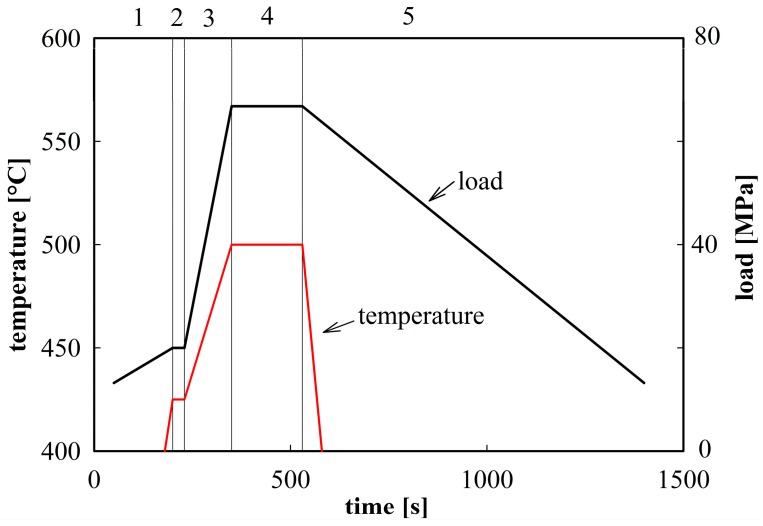
The scheme of the sintering process [11]: 1—set vacuum, heating to 425 °C, applying initial load 20 MPa; 2—temperature stabilization (30 s); 3—increase in temperature and load (120 s); 4—holding at selected temperature and load 75 MPa (180 s); 5—free cooling and parallel unloading.

The powder material was compacted in the spark plasma sintering device FCT SPS-HP25 using graphite die and punches. A sintering temperature of 500 °C was selected. The scheme of this sintering procedure is demonstrated in Figure 2. The SPS sintering produced cylindrical compacts with a radius of 25 mm and a height of 10 mm. The density of sintered compacts was estimated by He pycnometry to be 2800 kg·m^−3^ which is within the experimental scatter equal to the theoretical density of the Al7075 alloy (from 2800 to 2810 kg·m^−3^ [19]), so that a nearly fully dense material with relative density exceeding 99.6% was prepared.

The chemical composition of alloys designed as Al7075 can vary both in the content of main alloying elements (Zn, Mg, Cu) and in the content of impurities (especially Fe and Si). The chemical composition of our compact material after SPS was determined using energy dispersive spectroscopy and is given in Table 1.

The phase composition of the powder and the sintered material was verified by X-ray diffraction (XRD) using a diffractometer D8 Discover (Bruker AXS, Brno, Czech Republic) in Bragg-Brentano geometry with CuKα source and NiKβ radiation filter. Quantitative Rietveld analysis was performed by TOPAS V5 (Bruker AXS) to determine the weight fraction of all identified phases. The morphology of powder particles and microstructure of both the powder and the sintered materials were investigated on metallographically polished samples using a light microscope (LM) Olympus GX51 (Olympus, Prague, Czech Republic). Dix-Keller reagent was applied for 5 s to reveal the internal microstructure. Finer microstructural details were investigated by scanning electron microscopy (SEM) (FEI Quanta 200F, FEI, Brno, Czech Republic) using back scattered electrons (BSE). The scanning electron microscope equipped with EDAX Sapphire detecting unit for energy dispersive X-ray spectroscopy (EDS) (PV7760/77-ME, EDAX, Nachod, Czech Republic) and DigiView detector (1612, EDAX, Nachod, Czech Republic) for electron backscatter diffraction (EBSD) was used. The OIM software was employed for EBSD analysis. The preparation of samples for SEM included mechanical grinding, mechanical polishing up to 1 μm, chemical polishing with SiO_2_ oxide polishing suspension, and electro-polishing (just in case EBSD). Sample porosity was evaluated by image analysis of SEM images. Ten images of each polished sample at 800× magnification were taken and analyzed to fully represent the homogeneous sample microstructure.

The strength of materials was characterized using microhardness measurements. An automatic microhardness tester Qness Q10A+ (Qness GmbH, Golling, Austria) with a Vickers indentor was used. For the powder material, one indent with the load of 10 N was applied to an individual particle and the mean microhardness value was calculated from measurements performed at different powder particles. For the compact material, indentations were performed with an applied load of 50 N on planes parallel to the direction of the applied stress during SPS. In order to obtain good statistics, the areas of 6 × 6 mm^2^ were investigated with a distance of 200 µm between individual indents. The duration of such microhardness tests on each sample was about 10 h. Compression tests at room temperature were performed using an INSTRON 5582 testing machine (Instron, Canton, MA, USA).

## 3. Results

**Figure 3:**
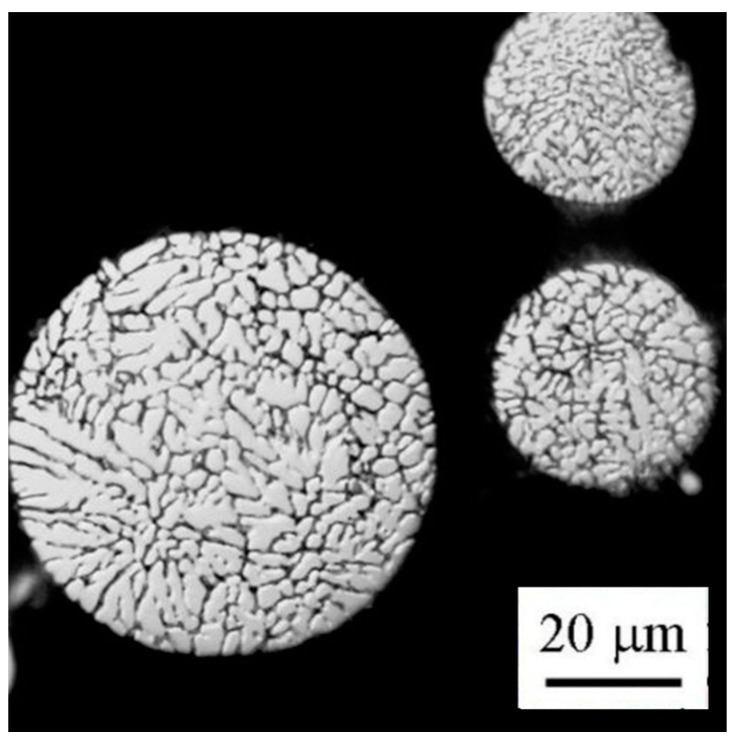
Solidification microstructure of atomized powder particles, LM.

**Figure 4:**
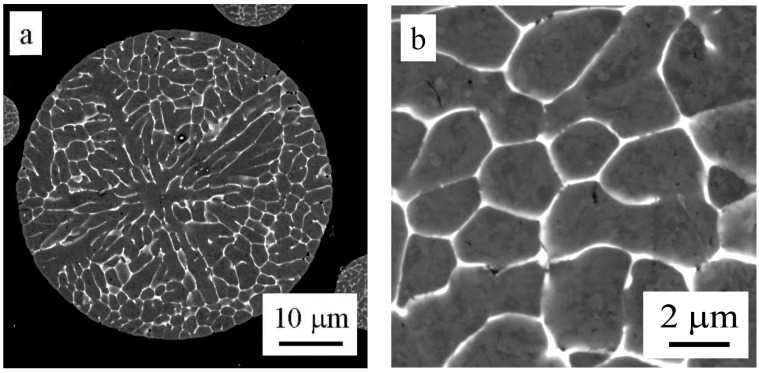
Solidification microstructure of atomized powder particles, SEM, (**a**) overview; (**b**) detail of a boundary region, BSE.

**Figure 5:**
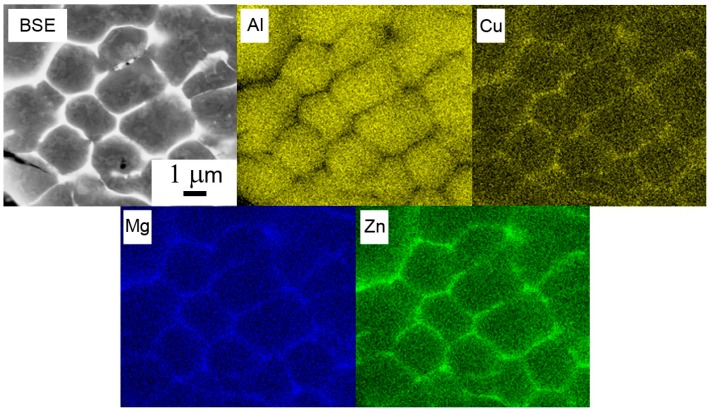
Element distribution in boundary regions of the atomized powder particle, BSE and the corresponding EDS maps.

Light microscopy revealed that almost all particles have a spheroidal shape. Finer particles generally exhibit a finer solidification microstructure (Figure 3). A combination of cellular and dendritic-like morphology was observed in individual powder particles using SEM (Figure 4a). The size of cells or dendrites lies in a range typically between 1 and 10 µm. The alloying elements are strongly segregated and form frequently continuous layers of intermetallic phases along boundaries (Figure 4b). The EDS experiment confirmed the presence of Mg, Zn, and Cu in boundary regions (Figure 5).

**Figure 6:**
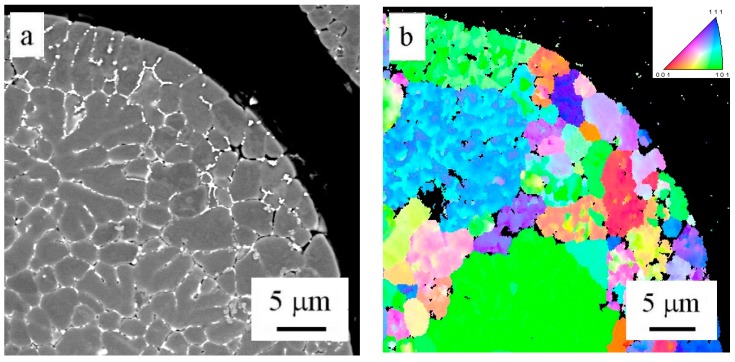
Solidification microstructure of atomized powder particles: (**a**) BSE contrast; (**b**) EBSD micrograph.

The EBSD method was used to determine the misorientation between areas divided by layers of intermetallic phases in the powder particle interior. Figure 6a shows a powder particle in BSE contrast and Figure 6b shows the corresponding EBSD micrograph. The comparison reveals that only some of the individual cells or dendrite-like formations can be considered as individual grains separated by high-angle boundaries. The size of grains varies from several micrometers up to more than 20 µm.

**Figure 7:**
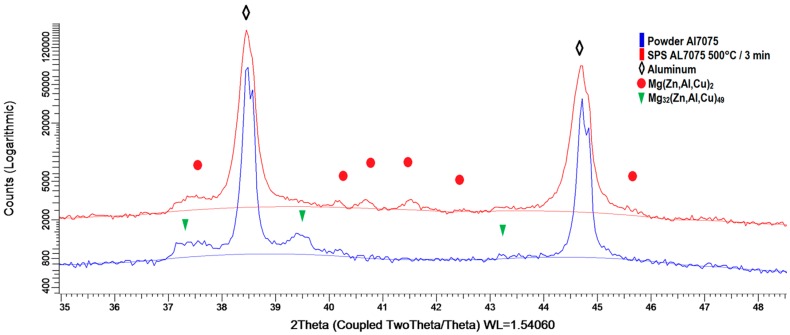
A part of the XRD pattern for the gas atomized Al7075 powder and its SPS compact sintered at 500 °C.

Figure 7 documents the phase composition estimated using XRD. The atomized powder contains predominantly the Mg_32_(Zn,Al,Cu)_49_ phase [20] with the weight fraction below 1 wt %. The fraction of this phase decreased at the expense of the Mg(Zn,Al,Cu)_2_ phase during sintering. The weight fraction of this new phase is about 1.7 wt % in the sintered material.

**Figure 8:**
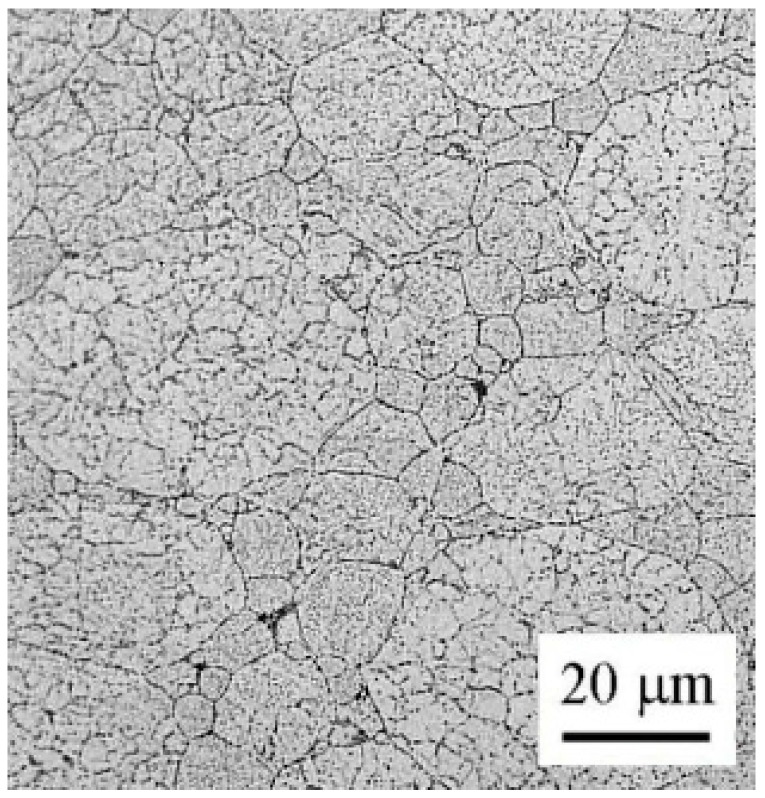
The microstructure of the material sintered at 500 °C, LM.

The microstructure of the SPS compact is shown in Figure 8. Both the boundaries of original powder particles and their internal microstructure are visible after sintering. The originally spherical shape of the powder particles is slightly changed to ellipsoidal as a result of plastic deformation during SPS.

**Figure 9:**
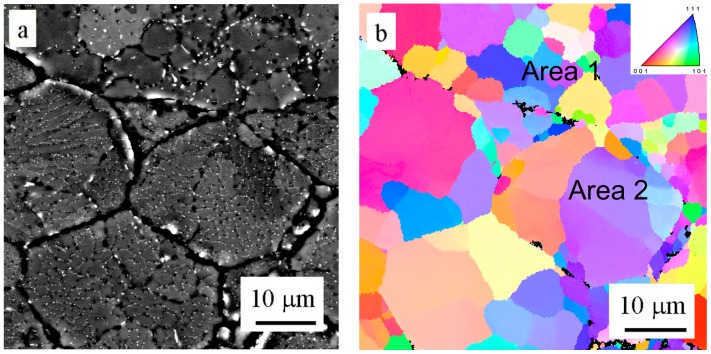
The microstructure of the material sintered at 500 °C, (**a**) BSE contrast; (**b**) EBSD micrograph.

A more detailed insight into the internal microstructure was obtained using SEM. Figure 9a reveals a change in the distribution of precipitates in the sintered material. The boundaries of original powder particles are decorated by relatively coarse precipitates of about 1 μm. The continuous layers of intermetallic phases observed in the original atomized powder along cell or dendrite boundaries are replaced by fine individual precipitates (from 150 to 200 nm). The image analysis revealed the volume fraction of pores between 0.2% and 0.5%.

The EBSD investigation (Figure 9b) revealed that former cells keep their original size and are separated predominantly by high-angle boundaries (Area 1). On the other side, the former dendrites coalesce into larger grains containing eventually low-angle boundaries (Area 2).

Microhardness measurement makes it possible to compare strength characteristics of powder and sintered materials. The Vickers microhardness value measured on atomized powder particles was 95 ± 18 HV. The large standard deviation was caused by the fact that each microhardness value was obtained on a different powder particle which might solidify at a different rate and therefore has a slightly different microstructure. The sintered material stored at room temperature for a long time exhibited a microhardness value of 150 ± 3 HV. No microhardness gradients were observed, either along the direction of applied stress or in the perpendicular direction. The compression tests performed on the sintered material stored at room temperature for a long time revealed an ultimate strength of 550 MPa and a plasticity of 25%.

The Al7075 alloy is a typical precipitation strengthened material and its microhardness depends on the thermal history. Isochronal annealing of the SPS compact for 1 h was applied in the temperature range from 150 to 500 °C and microhardness measurements were performed immediately after annealing and metallographic sample preparation at room temperature. The results are given in Table 2.

**Figure 10:**
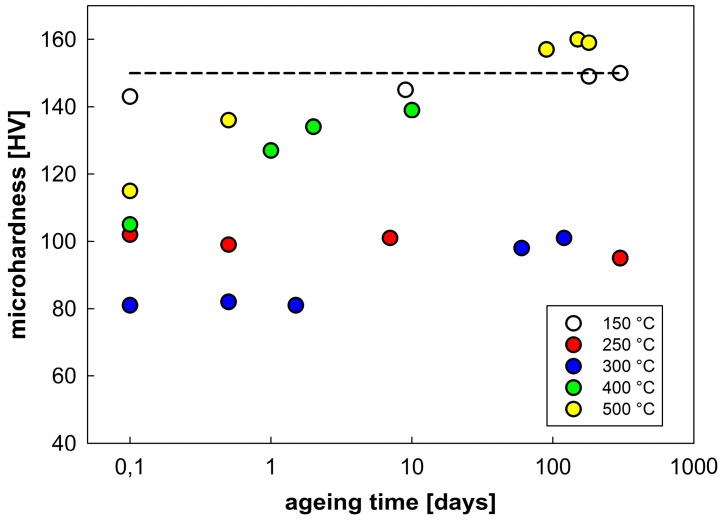
The influence of natural ageing at room temperature on microhardness of samples previously isochronally annealed at various temperatures for 1 h.

It follows from this table that annealing at temperatures up to 175 °C does not vary the microhardness in comparison with the as-sintered material. A decrease in microhardness starts after annealing at about 200 °C and its minimum values were found in samples annealed at 300 to 350 °C. An increase in microhardness was observed in samples annealed at higher temperatures. In order to understand this strange increase, the possible effect of natural ageing at room temperature was considered. The annealed samples were stored at room temperature and their microhardness was repeatedly measured after a stay at room temperature for various periods (Figure 10). Whereas the microhardness values of samples annealed at 150 and 250 °C do not exhibit significant changes during storage at room temperature even after more than nine months a slight increase was observed after about three months in the sample annealed at 300 °C. A fast increase in microhardness was observed in samples annealed at temperatures T ≥ 400 °C. The microhardness values measured at the sample annealed previously at 500 °C return to the value typical for non-annealed sintered material (indicated by a dashed line).

**Figure 11:**
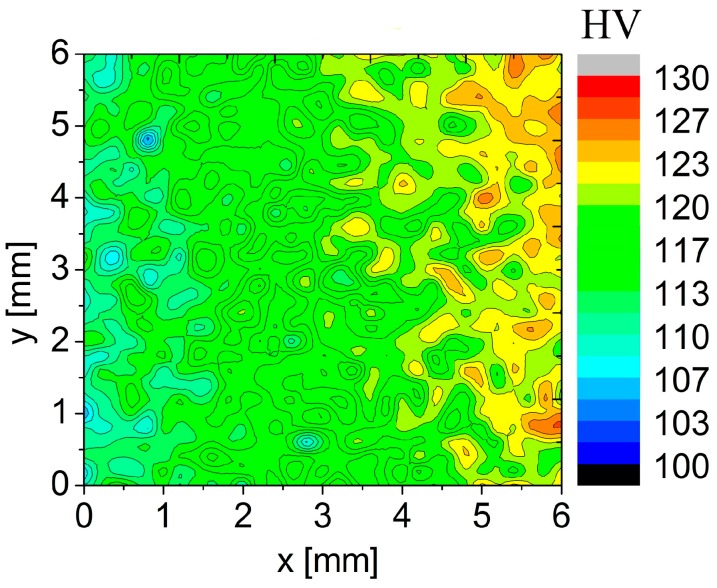
The evolution of microhardness during measurement of the sample previously annealed at 450 °C for 1 h.

The high rate of natural ageing in the sample previously annealed at 450 °C is documented by the microhardness map in Figure 11. The measurement started at the left side of the area and the observed starting microhardness values were close to 110 HV. The microhardness increased already during the measurement which lasted about 10 h. The gradual color gradient corresponds to the microhardness increase up to 125 HV.

**Figure 12:**
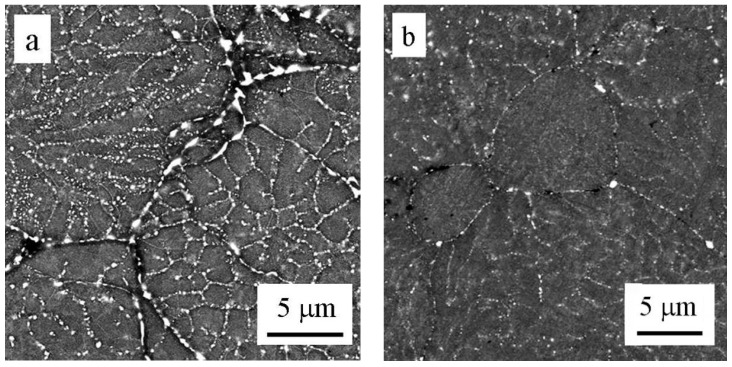
SEM/BSE micrographs of samples after annealing at (**a**) 250 °C for 1 h and (**b**) 500 °C for 1 h.

The observed microhardness variations reflect the changes in the microstructure, predominantly in the volume fraction, size, and distribution of precipitates. The BSE micrographs of samples annealed at 250 and 500 °C are shown in Figure 12. Well developed and relatively coarse particles were found in the sample after annealing at 250 °C. The coarser particles are located at boundaries of the original powder particles, slightly finer particles formed at internal boundaries of cells or dendrites. Much lower volume fraction and size of particles was observed in the sample annealed at 500 °C.

**Figure 13:**
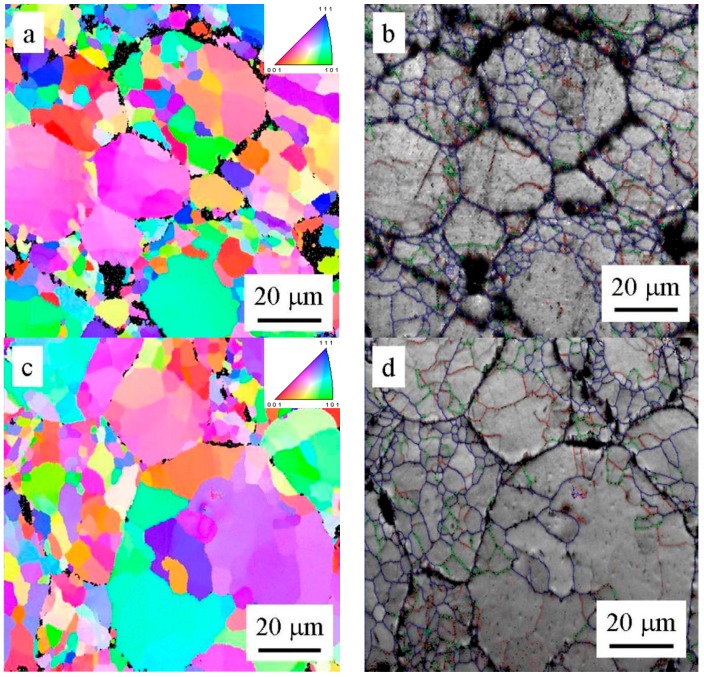
EBSD orientation maps (left) and distribution of grain boundaries (right) in typical areas of samples annealed at (**a**,**b**) 250 °C and (**c**,**d**) 500 °C boundaries with misorientation angles above 15° are blue, boundaries between 5° and 15° are green, and boundaries between 2° and 5° are red.

**Figure 14:**
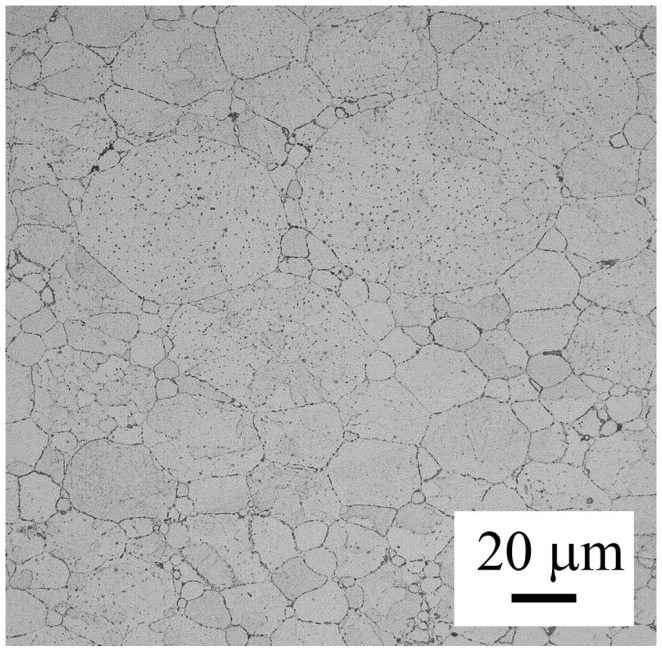
The microstructure of the material annealed at 500 °C, LM.

The influence of annealing at 250 and 500 °C on the grain size and character of grain boundaries was investigated using EBSD (Figure 13). The microstructure of both samples is similar—the original powder particles can be clearly distinguished. Some of the original powder particles are divided into a number of smaller grains separated by high-angle grain boundaries, others contain numerous low-angle boundaries. The fraction of high-angle boundaries with disorientation exceeding 15° is slightly above 50% in both samples. The numerical evaluation reveals a bimodal distribution of grain sizes with the first maximum at about 5 μm and the second one at about 30 μm. The grains smaller than 10 μm cover about 60% of the examined area in the sample annealed at 250 °C and about 45% in the sample annealed at 500 °C. The arrangement of former powder particles with various sizes is documented in Figure 14.

## 4. Discussion

Gas atomization is a typical representative of rapid solidification techniques. A high temperature gradient in the liquid phase and a high rate of solidification front movement are typical for the onset of solidification and can result in a featureless (segregation free) microstructure. Nevertheless, such microstructure can be formed either only in very small droplets or at the surface of larger droplets. During ongoing solidification both the temperature gradient and the rate of solidification front decrease and a breakdown of planar solidification front leads to the formation of dendritic or cellular microstructures. The solidification microstructure thus depends not only on the size of droplets but also on the position within a droplet [21]. Gupta et al. [22] studied a nitrogen atomized Al–Ti alloy and observed segregation free microstructure in droplets with the diameter bellow 10 μm, cellular morphology in droplets between 10 and 100 μm, and dendritic morphology in larger droplets. Similarly, Devaraj et al. [23] observed a microcellular structure only in very small particles of an atomized Al–4.5 wt % Cu alloy. As the particle size increased, the solidification microstructure changed to dendritic morphology. A similar solidification microstructure was observed in our Al7075 alloy. Featureless (segregation free) microstructure was not found at all and a combination of prevailing cellular and less frequent dendritic-like morphology was observed. The increasing powder particle size supported dendritic-like morphology. Similar results were reported by Rokni et al. [9].

The atomized materials are characterized usually only by a prevailing morphology type of solidification microstructure and a limited attention is paid to the character of interfaces between cells or dendrites. Saller et al. [24] studied the atomized powder particles of two Al–Fe alloys using the EBSD method and found that powder particles with a diameter about 10 μm were polycrystalline. Smaller grain sizes (close to 1 μm) and higher fraction of high-angle boundaries were observed in the low alloyed material where Fe was segregated along boundaries. Grain size exceeding 10 μm and a large number of low-angle boundaries were observed in the high alloyed material where the intermetallic phases were present. The atomized powder particles of our Al7075 alloy contain both regions with cells separated by predominantly high-angle boundaries and dendritic-like regions with numerous small-angle boundaries. The size of powder particles seems to be decisive—smaller powder particles exhibit a smaller grain size and a higher fraction of high-angle boundaries.

The main advantage of the powder metallurgy processing route is the possibility to produce near-net-shape compacts and to minimize finish machining and thus reduce material loss. On the other side, the main problem in the particulate consolidation processes is to suppress porosity, to remove oxide layers, and to retain all advantages resulting from rapid solidification. The conventional way of producing compact material from rapidly solidified Al-based particulates is a combination of cold pressing, degassing, hot consolidation at temperatures below degassing temperature, and a final hot working step (e.g., hot extrusion). The long times of exposure to high temperatures result in coarsening of the rapidly solidified microstructure and in approach to equilibrium state. Extremely elongated grains were observed in hot-extruded bars of the Al–Zn–Mg–Cu alloy [25] and a large number of low-angle boundaries was found in an Al7075 alloy modified by the addition of Zr [8].

The nature of electrical field/current interaction with powder particles in SPS is not clear yet and a variety of models can be found in literature (e.g., summary in [26]). A large Joule heat evolved at the contact points can lead to localized melting, enhancing interparticle bonding [27]. Additionally, effects of local electric and magnetic fields (e.g., local electrical discharges between individual powder particles) activate particle surface, which can significantly help to remove oxide layers and promote full densification. The steep temperature gradient along the particle radius causes the powder particle interiors to remain nearly unaffected. This benefit was observed also in our material—the structure is without coarse pores and the grain size along with the character of internal interfaces in SPS compacts remained unchanged in comparison with atomized powder particles (Figure 8). New high-angle boundaries are formed between original powder particles during SPS.

Several studies on Al-based materials revealed that increasing temperature of SPS promotes better densification of the powder material (e.g., [28,29]). A temperature of 600 °C was necessary in the microcrystalline Al of commercial purity to suppress porosity [30], 540 °C was found to be a suitable SPS temperature for the same material with coarser droplet size [31]. Our unpublished results revealed that very high density was achieved already after SPS at 425 °C. The different sintering response may be connected with the thickness of oxide layers on the surface of original powder particles or with a broader distribution of particle sizes in our powder material. Liu et al. [29] covered the nanocrystalline aluminum powder with an organic layer that prevented formation of oxides on the powder particle surface. The protected powder sintered successfully already at 450 °C. No systematic study of oxide layers was performed in our material. Their presence can be nevertheless indirectly deduced from results of mechanical tests where fracturing was observed at surfaces of original powder particles during bending tests [20] or high temperature tensile tests [32].

Devaraj et al. [23] revealed that the rate of punch movement during sintering was not constant and exhibited several maxima. The first maximum at the beginning of sintering was interpreted as a result of rearrangement of particulates caused by applied stress when small particulates filled in the space between the coarse ones. This stage is very important for the elimination of pores in SPS compacts, and it can be concluded that sintering of a material with a broader distribution of particulate sizes might be easier than in the ‘single-sized’ powders. This can be the case of our material and, consequently, a lower temperature of SPS is thus sufficient for full densification.

The Al7075 alloy used in our research was sintered at 500 °C, i.e., at a temperature exceeding the solution temperature of this material. It can be therefore expected that intermetallic phases present in the powder material should dissolve during sintering. However, the sintering time is very short and the solute atoms cannot diffuse over a long distance. During slow cooling from sintering temperature, new precipitates of intermetallic phases can be formed at places where continuous layers of intermetallic phases were present in powder particles, predominantly at interfaces between cells. Discrete precipitates arranged into chains were observed in sintered materials in the place of continuous layers. Simultaneously, the intermetallic phases changed—the Mg(Zn,Al,Cu)_2_ phase replaced the Mg_32_(Zn,Al,Cu)_49_ phase present in the original powder material. New precipitates of larger size were formed at surfaces of original powder particles. The detailed EDS investigation of the microstructure [20] also revealed the presence of particles containing impurities like Fe and Si.

The investigation of thermal stability of our sintered material revealed that microhardness exhibited a typical ageing response [16,17] if measured instantaneously after annealing to suppress natural ageing. It can be assumed that during cooling from sintering temperature only a part of solute atoms forms precipitated. The rest remained in the supersaturated solid solution and precipitated during subsequent annealing. The maximum strengthening was observed after annealing at temperatures between 150 and 175 °C and the material was stable during the following long stay at room temperature. The corresponding values of microhardness (≤150 HV) found in our material are slightly below the peak values (≅160 HV) and achievable in ingot metallurgical Al7075 alloy after a classical T6 heat treatment [33,34]. This reflects an expected lower volume fraction of fine strengthening phases in our material. A decrease in microhardness starting after annealing at about 200 °C can be explained by overaging, i.e., by the precipitation of stable η-Mg(Zn,Al,Cu)_2_ precipitates and by their growth. This is well documented in Figure 12a where a high density of relatively coarse precipitates can be observed. This overaged material is also stable during the following stay at room temperature. Further increase in annealing temperature should result in dissolution of precipitates. This process is well documented in Figure 12b, revealing much lower density and size of precipitates after annealing at 500 °C. Nevertheless, the increasing concentration of dissolved solutes with increasing annealing temperature promotes natural ageing. Both Figure 10 and Figure 11 clearly document the fast natural ageing which can enhance microhardness to values exceeding those found in the as-sintered material stored for a long time at room temperature.

Contrary to phase composition, thermal treatment has a relatively small influence on the grain size and character of interfaces in the SPS material (Figure 13). During SPS, a relatively small deformation is introduced at high temperatures. No significant increase in dislocation density can be expected and no recrystallization occurs during annealing. Additionally, the grain growth is probably suppressed by oxide particles located at the surface of the original powder particles and by precipitates decorating the internal interfaces.

The grain size observed in the gas atomized and then spark plasma sintered material does not reach the sub-microcrystalline range and is thus not fine enough to contribute significantly to strengthening. The microhardness values and their variations during thermal treatment are therefore determined by changes in phase composition. However, the gas atomized powder can be further processed by high energy milling resulting in a significant microstructure refinement. Consolidation of such material by SPS is expected to retain this microstructure and to contribute (in combination with enhanced dislocation density and suitable ageing) to an increase in strength in comparison with ingot metallurgical counterparts. These experiments will proceed.

## 5. Conclusions

(1) The Al7075 compacts without significant porosity were prepared from gas atomized powder by spark plasma sintering.
(2) The microstructure of atomized powder particles is cellular or dendritic-like with the size of cells or dendrites in a range typically between 1 and 10 μm. Both high- and low-angle boundaries were observed. The alloying elements are strongly segregated and form continuous layers of intermetallic phases (predominantly Mg_32_(Zn,Al,Cu)_49_ phase) along boundaries.
(3) The microstructure morphology does not vary during SPS and no significant growth of original cells was detected. The originally continuous layers of intermetallic phases are replaced by discrete precipitates formed predominantly by the Mg(Zn,Al,Cu)_2_ phase.
(4) The SPS material exhibits a typical ageing response. The maximum microhardness close to 150 HV was found after annealing at 175 °C for 1 h. A decrease in microhardness occurs after annealing at temperatures between 200 and 350 °C due to a formation of coarse precipitates. Annealing at higher temperatures results in a dissolution of precipitates. Microhardness of these samples increases due to a rapid natural ageing occurring already during the microhardness test. The peak microhardness close to 160 HV was achieved after three months of natural ageing.
(5) The grain size in SPS compacts is very stable, neither recrystallization nor grain growth were detected, even after annealing at 500 °C.

## Figures and Tables

**Table 1:** The chemical composition of the studied material.

Element	Weight Percentage (wt %)
Zn	5.3
Mg	2.1
Cu	1.3
Fe	0.40
Si	0.65
Al	Balance

**Table 2:** Microhardness values after isochronal annealing for 1 h at various temperatures.

Temperature (°C)	Microhardness (HV)
150	140 ± 5
175	148 ± 6
200	132 ± 5
250	102 ± 4
300	81 ± 1
350	84 ± 3
400	105 ± 6
450	117 ± 4
500	115 ± 10
